# Chinese herbal medicines in the treatment of ulcerative colitis: a review

**DOI:** 10.1186/s13020-022-00591-x

**Published:** 2022-04-04

**Authors:** Xuan Zhang, Lin Zhang, Jacky C. P. Chan, Xihong Wang, Chenchen Zhao, Ying Xu, Weifeng Xiong, Wai Chak Chung, Feng Liang, Xu Wang, Jiangxia Miao, Zhaoxiang Bian

**Affiliations:** 1 Chinese Clinical Trial Registry (Hong Kong), Hong Kong Chinese Medicine Clinical Study Centre, Chinese EQUATOR Centre, School of Chinese Medicine, Hong Kong Baptist University, Hong Kong, Hong Kong, SAR of China; 2 Centre for Chinese Herbal Medicine Drug Development Limited, Hong Kong Baptist University, Hong Kong, Hong Kong, SAR of China; 3 Tianjin University of Traditional Chinese Medicine, Tianjin, China; 4 Department of Computer Science, HKBU Faculty of Science, Hong Kong Baptist University, Hong Kong, Hong Kong, SAR of China; 5 Oncology Department, The Second Affiliated Hospital of Tianjin University of Traditional Chinese Medicine, Tianjin, China; 6 College of Chinese Medicine, Beijing University of Chinese Medicine, Beijing, China; 7 School of Chinese Medicine, The Chinese University of Hong Kong, Hong Kong, Hong Kong, SAR of China

**Keywords:** Chinese herbal medicine formula, Clinical practice of Chinese medicine, Data mining, Literature review, Pattern identification, Ulcerative colitis

## Abstract

**Objective:**

To investigate how the ulcerative colitis (UC) be treated with Chinese herbal medicines (CHM), using Chinese medicine (CM) pattern (*zheng*) identification, in the current clinical practice.

**Methods:**

A total of 7 electronic databases were systematically searched for UC clinical studies with CHM interventions (including single herbs and CHM formulas) published in English and Chinese from the date of their inception to November 25, 2020. Descriptive statistics were adopted to demonstrate the characteristics of study design, and to collate the commonly CM patterns of UC and frequently used CHM herbs and formulas. Further, IBM SPSS Modeler 18.0 and Cytoscape 3.7.1 software were used to analyze and visualize the associations between different categories of CHM and their *zheng* indications.

**Results:**

A total of 2311 articles were included in this study, of which most (> 90%) were RCTs with CHM formulas. The most common *zheng* of UC was *Large intestine dampness-heat*, while the basic type of CM patten was *Spleen deficiency*. The most frequently used classical formula was *Bai-Tou-Weng-Tang*, followed by *Shen-Ling-Bai-Zhu-San*, and the commonly used proprietary CHM was *Xi-Lei-San* (enema). Sulfasalazine and Mesalazine are commonly used as concomitant western medicines. The most frequently used single medicinals were *Huang Lian* and *Bai Zhu*, which also identified as the core herbs for different CM patterns.

**Conclusion:**

This study examined the application of CHM interventions for UC and summarized their characteristics in clinical practice. These data indicated there were limited information about the safety assessment of CHM formulas and further RCTs including CM pattern(s) with strict design are necessary.

**Supplementary Information:**

The online version contains supplementary material available at 10.1186/s13020-022-00591-x.

## Background

Ulcerative Colitis (UC), which is one type of inflammatory bowel disease (IBD, another is Crohn’s Disease), is a chronic, progressive and disabling condition [1]. The clinical manifestation of UC mainly includes mucus bloody stool, recurrent diarrhea, and abdominal pain [2]. As a lifelong disease, the symptoms of UC can lead to a substantial negative impact on patient quality of life and incur a significant economic burden, including both direct medical costs and indirect costs associated with absenteeism and productivity loss [3]. The etiology of UC, however, remains not well identified and it may result from environmental factors, genetic predisposition, microbial dysbiosis, and immune dysregulation [4, 5]. The incidence of UC, a disease which previously more prevalent in high-income countries of Europe and North America has shifted towards industrialized countries such as Asia [6]. As a result, it has become a global refractory disease with worldwide shifting epidemiological characteristics [7].

There is no successful therapy that can treat UC so far, even surgery may be followed by ongoing morbidity [8]. Currently, the optimal strategy of management includes remission maintenance, prevention of disease-related complication, improvement of health-related quality of life and promotion of mucosal healing [9]. Pharmacological therapies, including 5-aminosalicylic acid, corticosteroids, immunosuppressants, biological agents, and other promising treatments (e.g., fecal microbiota transplantation) have been continuous developed, but some patients may experience gradual loss of response to the therapy while others may show intolerance to adverse effects of drugs, such as allergic reactions, digestive tract symptoms (e.g., flatulence, abdominal pain, nausea, and diarrhea), headache, and hypertension [10, 11]. As a result, an increasing number of UC patients (ranging between 21 and 60%) seek help from complementary and alternative medicine (CAM), especially Chinese medicine (CM) therapies, which has been practiced in Asia for thousands of years [12, 13].

As the most common interventions in CM practice, Chinese herbal medicines (CHM) have been widely used for UC due to the unique advantages of efficacy and safety [14]. An increasing number of evidence have shown that CHM have potentially positive effects on the relief of abdominal pain, diarrhea, and inflammation [15–17]. In CM theory, CHM interventions include Chinese medicinal substances (single herbs) and CHM formulas (namely “*Fu-Fang*”, or specific combinations of generally more than two Chinese medicinal substances). Since disease is a dynamic process, there may be different patterns in different phases of a disease. The success in deciding on a CHM prescription depends on an accurate pattern (*zheng*) diagnosis known as *Bian-Zheng-Lun-Zhi* (e.g., treatment based on pattern differentiation) [18]. However, previous studies published regarding the effectiveness of CM interventions for UC paid little attention to the application of pattern identification [19, 20]. In addition, we found that there is no latest review so far to provide the current clinical practice of CHM used for UC and its characteristics in study design [21].

This study aimed to investigate CHM applications for UC, especially based on CM pattern identification. The objectives were as follows: (1) to summarize the general characteristics of published UC clinical studies with CHM intervention(s); (2) to review the most frequently used CHM herbs and formulas, and the common types of CM patterns in UC; (3) to analyze the herbal correlations based on different pattern indications. The review will determine the current practice of CHM treatments for UC, and also identify the common problems (if any) in the previous research. These results will provide the basis for further studies regarding CHM based on pattern identification for UC.

## Methods

### Inclusion and exclusion criteria

This study included clinical studies of UC with CHM intervention(s) published in English and Chinese up to November 25, 2020. We included studies on subjects given the diagnosis of UC defined by clear diagnostic criteria or references, regardless of age, gender, course of disease and severity. The CHM interventions are typically administered as either single herbs or formulas (e.g., fixed or individualized formula, patent proprietary formula), which may have been administered alone or in combination with other interventions of conventional Western Medicine (WM) or CAM. No limitation on their formulation, preparation, dosage, dosage form or route of administration. The control intervention could be active, conventional medicine, placebo, no treatment, or other CAM treatments, etc. There were no limitations in the assessed outcomes. We considered randomized controlled trials (RCTs), quasi-randomized controlled trials, non-randomized controlled trials, observational clinical studies (e.g., cohort study or cross-sectional study) including the control group(s), case–control studies for inclusion. We excluded the following studies: repeated and withdrawal publications, studies with non-CHM interventions, comprehensive interventions focused on pharmacological treatment (or other CAM treatment) rather than CHM, plant extracts (e.g., plant-derived chemicals or synthetic chemicals which contain constituents of plants), clinical studies without a comparison or control group(s), study protocols, reviews, case series, case reports, abstracts or full-text reports not found, non-human studies, and non-English or non-Chinese language reports.

### Search strategy

The following 7 databases, EMBASE, MEDLINE, Evidence-Based Medicine (EBM) Reviews, Allied and Complementary Medicine (AMED), The China National Knowledge Infrastructure (CNKI), VIP database, and Wanfang database were searched from the date of their inception to November 25, 2020. Languages were restricted to English and Chinese. The search terms were “ulcerative colitis”, “inflammatory bowel disease”, “Chinese medicine”, “Chinese materia medica”, “herbal formula”, “controlled trial”, “randomization”, and “clinical trial”, etc. The detailed search strategy is given in Additional file 1: Appendix S1. It is noted that the trial registration platforms (e.g., ClinicalTrials.gov) are not included in our search databases because (i) the records of some completed trials were duplication with their final publications, and (ii) some ongoing trials presented inadequate or absent details of CHM interventions which cannot meet the requirements of data collection in this review.

### Screening

The titles and abstracts of the records were independently screened by two researchers (XHW and YX) based on inclusion and exclusion criteria, and the full texts of potentially suitable articles were retrieved for further assessment. Disagreements, if any, were resolved by discussion or consultation with the third researcher (XZ).

### Data extraction

For each study, the following information was collected: article title, year of publication, study design and setting, sample size, clinical phases of UC, CM patterns (if any), CM treatment principles (if any), types and details of CHM interventions (including name, composition, preparation, dosage, dosage form, and administration route), integrative application of CHM and WM drugs (if any), treatment regimen (including duration of intervention and follow-up period), categories of the controls and outcomes, and safety evaluation. Each item was extracted by two researchers independently. Before extraction, they received a data extraction form and trained by an experienced researcher (XZ) to understand the criteria for each item. A third researcher (LZ) checked the extracted data based on Visual Basic for Applications (VBA) and made corrections (Additional file 1: Appendix S2). Any uncertainty regarding a particular data were resolved by consultation with the principal investigator (ZXB).

### Data standardization

Due to a variety of expressions for the similar/same CM patterns and treatment principles, we standardize the data as a uniform and internationally recognized terminology which is easier for categorization and calculation. For example, we classified the phrases of “draining dampness”, “drying dampness”, “eliminating dampness”, “resolving dampness”, “dampness-dispelling” as one treatment principle. Also, we grouped pattern/syndrome of “dampness obstruction”, “dampness accumulation”, “dampness immersion”, and “dampness steaming” as the same type of CM pattern. All Chinese-to-English translations of terminology were deduced primarily from the international standards of “WHO International Standard Terminologies on Traditional Medicine in the West Pacific Region” and/or the “International Standard Chinese-English Basic Nomenclature of Chinese Medicine”, which were published by World Federation of Chinese Medicine Societies in 2007 [22–24]. The name of CHM interventions (including the herbs and formulas) was translated according to the Chinese Pharmacopoeia 2020 [25].

### Statistical analysis

(1) Descriptive statistics: All data were collected and recorded in Microsoft Office Excel (Version 2016). Descriptive data were presented as number (n) and percent (%), or mean and standard deviations (SD). Data analyses were performed using SPSS software, version 25.0. (2) Association Rule Mining (ARM): The association rule, a well-recognized data mining and knowledge discovery approach, has been widely applied in medical areas. Based on IBM SPSS Modeler 18.0 software, the CHM herbs and formulas were analyzed by frequency, clustering and high-frequency herb using association rule, e.g., Apriori algorithm, to attempt to find rules and associations that exist between the prescriptions and pattern identifications.

Specifically, based on the descriptive data regarding to the frequency of herbs and formulas in the treatment of UC with different CM patterns, we used Apriori algorithm to determine the commonly used herb group (two or three-herbs combinations) and network analysis to determine the correlation of different herbs. The strength of an association rule could be measured by its support and confidence. We used the support level to determine the probability that A and B occurred simultaneously. The support of the item set A: s(A) = σ(A)/N; support for rule A ⟶ B: s (A ⟶ B) = σ(A ∪ B)/N. We used confidence to determine how frequently B occurred in transactions containing A: c (A ⟶ B) = σ(A ∪ B)/σ(A). The minimum rule confidence level was set as 80%, which indicated when A herb appeared, the appearance of B herb would be 80%, and the minimum conditional support was set to 20%. In addition, we used the lift to observe whether the two item sets were dependent on each other, for which value larger than 1 indicated the two item sets depended on each other [26]. After data preprocessing and correlation analysis between herbs, a directed network was constructed to connect herbs that were used together to summarize prescriptions based on different CM patterns. We converted the text information in the data source to a vector form, marked the annotation name herbs as the feature vector, and input them to the 0/1 structured herbs prescription table. Then, the table was imported into IBM SPSS Modeler 18.0 to create association rules flow, which could output the associated node herbs and their weight values. According to the frequency correlation between various CHM herbs, the IBM SPSS Modeler 18.0 software was used to classify the frequency into three types of correlation degree, including weak, between weak and strong, and strong link. We filtered a total of sixteen herbs in each CM pattern, which presented a strong association with the core herb (e.g., frequency more than 20). Then, we numbered the output nodes with strong associations created a list of the combination of nodes list and edges list, which was imported into Cytoscape 3.7.1 software to perform network image visualization.

## Results

### Search

**Fig. 1:**
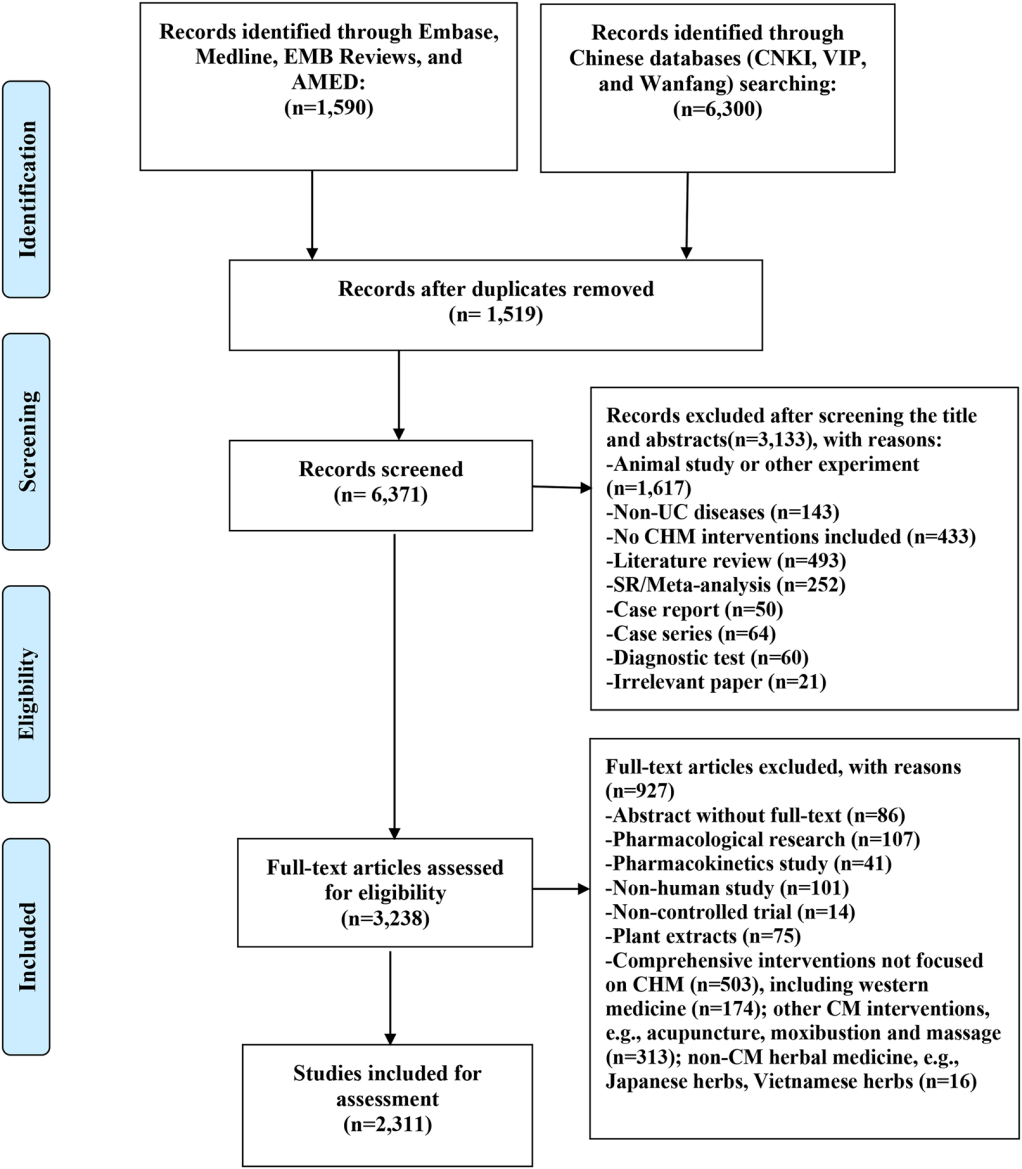
Flow chart of the search and selection process

The initial search identified a total of 7890 records, of which 3238 relevant studies were retained after excluding duplicates and screening the titles and abstracts. After reviewing the full text of these publications, we identified 2311 articles for inclusion in our final analysis (Fig. 1).

### Characteristics of included studies

**Fig. 2:**
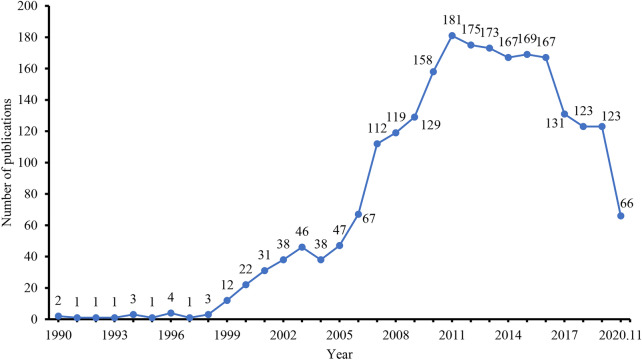
Number of CHM interventions studies for UC included from 1990 to November 2020

The earliest clinical study of UC with CHM intervention included in this study was published in 1990. The total number particularly increased after 2007, reaching more than 100 per year and peaking during 2011–2016 (e.g., average number was 172 each year) (Fig. 2). The characteristics of included trials are shown in Table 1. Most included studies are RCT (93.6%) with parallel two arms (96.4%), but few included multicentre (2.9%) and blinding (3.0%) design. Sample size ranged from 10 to 2568, median 78 (inter-quartile range 60–100). Commonly, the clinical studies focused on active UC (71.2%), CHM formula interventions (99.1%) and active control (54.9%). There were 895 (38.7%) studies that considered CM pattern in the inclusion of UC participants, but 29.1% of them did not provide the diagnosis criteria. The fixed formula (58.1%) and decoction (87.2%) were reported in a majority among CHM formulas. Further, 38.6% CHM formulas were combined with Western medicine(s) in the application of clinical practice. In terms of administration route of CHM therapies for UC, external delivery (41.2%) is more common than oral route (34.9%). Few trials included CM-related indicators (22.7%), such as CM pattern scale questionnaire, and safety assessment (30.2%) in the outcomes. Among 697 studies that considered the adverse effect (AE) of CHM interventions, 67% identified no AEs while 33% reported the specific AEs (Additional file 1: Appendix S3).

**Table 1:** Characteristics of included studies (n = 2311)

Characteristic	n (%)
Type of study	
Randomized controlled trial (RCT)^a^	2164 (93.6)
Others^b^	147 (6.4)
Trial participating centre	
Single centre	2243 (97.1)
Multicentre	68 (2.9)
Type of blinding	
Open label	2243 (97.1)
Single	34 (1.5)
Double	34 (1.5)
Sample size	
Median and IQR (inter-quartile range)	78 (60–100)
Number of assigned groups	
2	2228 (96.4)
3	73 (3.2)
> 3	10 (0.4)
Clinical phases of UC	
Active^c^	1645 (71.2)
Remission	636 (27.5)
Both	30 (1.3)
UC with CM pattern (*zheng*)	
Yes	895 (38.7)
No	1416 (61.3)
CM pattern with a diagnosis criteria basis or reference^d^	
Yes^e^	635 (70.9)
No^f^	260 (29.1)
Type of CHM interventions	
Chinese single herb(s)	20 (0.9)
CHM formula(s)	2291 (99.1)
Type of CHM formulas^g^	
Fixed CHM formulas	1332 (58.1)
Individualized CHM formulas	817 (35.4)
Patent proprietary CHM formulas	191 (8.3)
Dosage form^h^	
Decoction	2015 (87.2)
Granule	71 (3.1)
Powder	71 (3.1)
Capsule	61 (2.6)
Enema	41 (1.8)
Others^i^	124 (5.4)
Administration route	
Oral	806 (34.9)
External	951 (41.2)
Both	554 (24.0)
Administration time of CHM intervention (weeks)	
Median and IQR (inter-quartile range)	6 (4–9)
Follow-up period (weeks)^j^	
Median and IQR (inter-quartile range)	26 (21–52)
Treatment group	
Only CHM intervention(s)	1418 (61.4)
CHM combined with Western medicine(s)	893 (38.6)
Type of controls	
Active control	1268 (54.9)
Add on control with baseline treatment	735 (31.8)
Placebo control	34 (1.5)
Blank or waitlist control	4 (0.2)
Others^k^	270 (11.7)
Outcomes including CM-related indicators	
Yes	524 (22.7)
Outcomes including safety assessment	
Yes	697 (30.2)
No	1614 (69.8)
Adverse effects (AE) reported^l^	
No AE was identified	467 (67.0)
Specific AE was reported^m^	230 (33.0)

^a^Including both randomized and quasi-randomized controlled trials

^b^Including non-randomized controlled trials, controlled observational studies, cohort study, cross-sectional study, and case–control studies

^c^ Including mild, moderate, and severe active UC

^d^ The percentage of this item was based on the number of records that included CM pattern diagnosis (n = 895). For example, 70.9% = 635/895

^e^ Reporting of how the CM pattern is diagnosed and what criteria are used for including and excluding participants, such as specific description or reference(s) where detailed explanation(s) can be found

^f^ Only provided the name(s) of CM pattern without any diagnosis criteria

^g^ Some trials included more than one type of formulas, such as both individualized formula and patent proprietary formula were included; these were counted in different columns. Thus, the total number of formula types was above 2,291. But the total number of articles that studied CHM formulas was 2,291, therefore, the percentage of each sub-item was based on 2,291

^h^ Some trials included more than one CHM interventions with different dosage forms; these were counted in different columns. Thus, the total number of dosage forms was above 2,311. We listed the top five in the Table, and categorized the remaining dosage forms into the column of “Others”

^i^ Including Pill (32), Plaster (28), Suppository (25), Tablet (23), Injection (9), Cataplasm (4), and Gelatin (3)

^j^ Among 2,311 studies, only 492 (21.3%) articles reported the follow-up arrangement

^k^The control group included other CHM prescription(s), such as common CHM formula, fixed CHM formula, or patent proprietary CHM formula, etc. Besides, different doses or dosage form of CHM formulas were also included as the control

^l^ The percentage of this item was based on the number of articles that included safety assessment (n = 697). For example, 70.8% = 635/897

^m^ The specific AEs were provided in Additional file 1: Appendix S3 with detailed symptoms and its reporting frequency

### Description of CHM treatments for UC

**Fig. 3:**
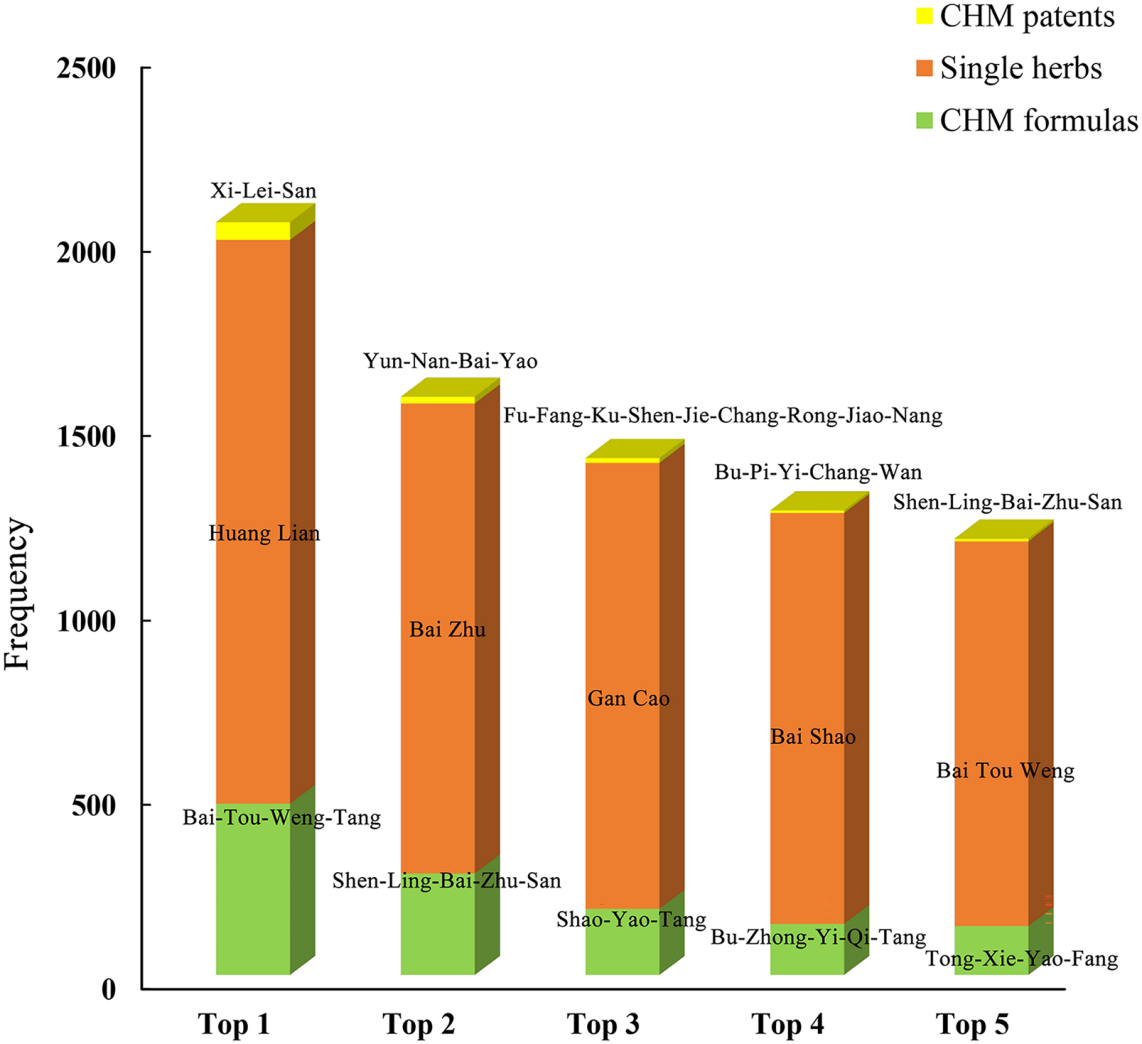
Top 5 frequency used CHM interventions for UC

Among 2311 included studies, we identified a total of 245 kinds of CHM formulas (Additional file 1: Appendix S4) and the most frequently used prescription was *Bai-Tou-Weng-Tang* (frequency = 466), followed by *Shen-Ling-Bai-Zhu-San* (frequency = 276), *Shao-Yao-Tang* (frequency = 180), *Bu-Zhong-Yi-Qi-Tang* (frequency = 139), and *Tong-Xie-Yao-Fang* (frequency = 133). Around 26.3% of these top five CHM formulas were commonly combined with western medicines, such as Sulfasalazine and Mesalazine, in clinical practice. Details of their compositions, actions and indications are summarized in Table 2. Moreover, the most common herb used in practice was *Huang Lian* (frequency = 1528), followed by *Bai Zhu* (frequency = 1275), *Gan Cao* (frequency = 1210), *Bai Shao* (frequency = 1115), and *Bai Tou Weng* (frequency = 1045) and so on. We listed the top ten frequently used single herbs in Table 3. The application of patent proprietary CHM formula is very convenient in practice, we thereby provided the top five commonly used proprietary CHMs in Table 4, including *Xi-Lei-San* (frequency = 48), *Yun-Nan-Bai-Yao* (frequency = 18), *Fu-Fang-Ku-Shen-Jie-Chang-Rong-Jiao-Nang* (frequency = 13), *Bu-Pi-Yi-Chang-Wan* (frequency = 7), and *Shen-Ling-Bai-Zhu-San* (frequency = 7). Together, we provided Fig. 3 to summarize the top 5 frequently used CHM interventions.

**Table 2:** Summary of top five most frequently used CHM formulas^a^

English name (Phonetic)	Combination (Phonetic)	Actions	Indications	Frequency of usage	Frequency of integration with Western medicine^b^
Pulsatilla Decoction *(Bai-Tou-Weng-Tang)*	*Pulsatillae Radix (Bai Tou Weng), Phellodendri Chinensis Cortex (Huang Bai), Coptidis Rhizoma (Huang Lian), Fraxini Cortex (Qin Pi)*	Clears heat, resolves toxins, cools the blood, and arrests dysentery	This formula is indicated for heat toxin bloody dysentery. The symptoms are stools with pus and fresh blood, abdominal pain, abdominal urgency with rectal heaviness, burning sensation in the anus, thirst with a desire to drink, a red tongue with yellow coating, and a slippery, rapid pulse	466	Mesalazine: 86 Sulfasalazine: 83
Ginseng, Poria and Atractylodes Macrocephala Powder *(Shen-Ling-Bai-Zhu-San)*	*Nelumbinis Semen (Lian Zi Xin), Coicis Semen (Yi Yi Ren), Amomi Fructus (Sha Ren), Platycodonis Radix (Jie Geng), Lablab Semen Album (Bai Bian Dou), Poria (Fu Ling), Ginseng Radix et Rhizoma (Ren Shen), Glycyrrhizae Radix et Rhizoma (Gan Cao), Atractylodis Macrocephalae Rhizoma (Bai Zhu), Dioscoreae Rhizoma (Shan Yao)*	Boosts qi and fortifies the spleen, drains dampness and arrests diarrhea	This formula is indicated for patterns of spleen deficiency with excessive dampness accumulation marked by epigastric bloating or stuffiness, borborygmus, diarrhea, lack of strength in the four limbs, thin body, and lusterless yellow facial complexion. The tongue is pale with a white, greasy coating and the pulse is moderate and deficient	276	Sulfasalazine: 38 Mesalazine: 19
Peony Decoction *(Shao-Yao-Tang)*	*Paeoniae Radix Alba (Shao Yao), Angelicae Sinensis Radix (Dang Gui), Coptidis Rhizoma (Huang Lian), Arecae Semen (Bing Lang), Aucklandiae Radix (Mu Xiang), Glycyrrhizae Radix et Rhizoma (Gan Cao), Rhei Radix et Rhizoma (Da Huang), Scutellariae Radix (Huang Qin), Cinnamomi Cortex (Rou Gui)*	Clears heat in the large intestine, dries dampness, invigorates the blood, and moves qi	This formula is indicated for damp-heat dysentery. The symptoms are dysentery with stool containing pus and blood, abdominal pain, abdominal urgency with rectal heaviness, burning sensation in the anus, scant dark urine, a greasy yellow tongue coating, and a wiry rapid pulse	180	Sulfasalazine: 26 Mesalazine: 21
Center-Supplementing and Qi-Boosting Decoction *(Bu-Zhong-Yi-Qi-Tang)*	*Astragali Radix (Huang Qi), Glycyrrhizae Radix et Rhizoma Praeparata cum Melle (Zhi Gan Cao), Ginseng Radix et Rhizoma (Ren Shen), Angelicae Sinensis Radix (Dang Gui), Citri Reticulatae Pericarpium (Chen Pi), Cimicifugae Rhizoma (Sheng Ma), Bupleuri Radix (Chai Hu), Atractylodis Macrocephalae Rhizoma (Bai Zhu)*	Supplements the center and boosts qi; raises yang and lifts the sunken	This formula is indicated for two patterns. The first pattern is deficient or sunken spleen qi with reduced food intake, general sluggish sensation, weak breathing, lack of desire to speak, sallow-yellow facial complexion, and loose unformed stool. The tongue is pale and the pulse is deficient. The second pattern is objective or subjective fever due to qi deficiency manifested by a feverish sensation, spontaneous sweating, thirst with a desire for hot drinks, shortness of breath, and lack of strength. The tongue is pale and the pulse is deficient, big, and weak	139	Sulfasalazine: 18 Mesalazine: 13
Important Formula for Painful Diarrhea *(Tong-Xie-Yao-Fang)*	Atractylodis Macrocephalae Rhizoma (*Bai Zhu*), Paeoniae Radix Alba (*Bai Shao*), Citri Reticulatae Pericarpium (*Chen Pi*), Saposhnikoviae Radix (*Fang Feng*)	Supplements the spleen and softens the liver, dispels dampness and arrests diarrhea	This formula is indicated for painful diarrhea due to a deficient spleen and a vigorous liver, characterized by borborygmus, abdominal pain, diarrhea with abdominal pain, pain that is relieved after diarrhea, the guan pulses of both hands not in harmony (a wiry pulse on the left hand and a moderate pulse on the right)	133	Sulfasalazine: 21 Mesalazine: 14

^a^ Including both fixed formulas and individualized formulas. Thus, the top five formulas provided in the Table also included their modifications

^b^ We selected the integrated western medicines which have been used more than 10 times

**Table 3:** Summary of top ten most frequently used Chinese medicinal substances (single herbs)

English name (Phonetic)	Latin Pharmaceutical Name	Properties	Functions	Quality Markers^a^	Frequency of usage
Chinese Goldthread Rhizome (*Huang Lian*)	*Coptidis Rhizoma*	Bitter; cold	Clears heat, dries dampness, drains fire, resolves toxin. Apply to damp-heat distention and fullness, vomiting and acid regurgitation, diarrhea, jaundice, dizziness with high fever, excess of heart fire, insomnia due to vexation, haematemesis and epistaxis due to blood heat, red eye, toothache, diabetes, abscess, sore and boil swelling, externally used to eczema, ear pus	Berberine	1528
Largehead Atractylodes Rhizome (*Bai Zhu*)	*Atractylodis Macrocephalae Rhizoma*	Bitter, sweet; warm	Fortifies the spleen, boosts qi, dries dampness, promotes urination, stops sweating, calms the fetus. Apply to deficiency-weakness of spleen, anorexia, and abdominal distension, and loose tools, dizziness induced by phlegm and retained fluids, edema, spontaneous perspiration, restless fetal movement	Atractylon, Atractylol	1275
Liquorice Root (*Gan Cao*)	*Glycyrrhizae Radix et Rhizoma*	Sweet; neutral	Supplements the spleen, boosts qi, clears heat, resolves toxin, dispels phlegm, relieves cough, relaxes tension, relieves pain, harmonizes the nature of other medicinals	Liquiritin, Glycyrrhizic Acid	1210
White Peony Root (*Bai Shao*)	*Paeoniae Radix Alba*	Bitter, sour, slightly cold	Nourishes the blood, regulates menstruation, calms the liver, relieve pain, astringes yin, stops sweating. Apply to thoracic, abdominal and costal pains, abdominal pain due to dysentery, spontaneous perspiration and night sweat, fever with yin deficiency, irregular menstrual periods, metrorrhagia and metrostaxis, leukorrhea	Paeoniflorin	1115
Chinese Pulsatilla Root (*Bai Tou Weng*)	*Pulsatillae Radix*	Bitter, cold	Clears hear, resolves toxin, cools the blood, relieves dysentery, eliminating dampness and destroying parasites. Apply to heat dysentery, epistaxis, hemorrhoid hemorrhage, leucorrhea, pudendum itch, ulcer and carbuncle, struma	Anemoside B4	1045
Common Bletilla Tuber (*Bai Ji*)	*Bletillae Rhizoma*	Bitter, sweet, astringent; slightly cold	Tonify the lung, stop bleeding, reduces swelling, engenders flesh, astringes core. Applied to empsyxis, non-traumatic hemorrhage, traumatic hemorrhage, anthracia and swelling caused by toxicity, pain due to ulcer, scalding and scorching injuries, rhagadia manus et pedis	Militarine	1027
Amur Corktree Bark (*Huang Bai*)	*Phellodendri Chinensis Cortex*	Bitter; cold	Clears heat, dried dampness, drains fire, eliminates steaming, resolves toxin, treats sore. Apply to dysentery due to damp-heat pathogen, jaundice, leukorrhea, pyretic stranguria, beriberi, osteopyrexia, night sweat, emissions, burns and scalds and swelling, eczema and pruritus	Berberine, Phellodendrine	945
Costusroot (*Mu Xiang*)	*Aucklandiae Radix*	Acrid, bitter, warm	Moves qi, stops pain, adjusts middle, resolves stasis, warms middle, digests food	Costunolide, Dehydrocostus Lactone	931
Mongolian Milkvetch Root (*Huang Qi*)	*Astragali Radix*	Sweet; warm	Supplements qi, secures the exterior, promotes urination, outthrusts toxin, expels pus, closes sore, engenders flesh. Apply to deficiency of vital energy causing debilitation, anorexia and loose stools, collapse of middle-warmer energy, anal prolapse due to long term diarrhea, hemafecia, metrorrhagia and metrostaxis, continuous persipiration due to superficial deficiency, persistent superficial infection, persistent ulcers, wilting due to blood deficiency, internal heat dispersion-thirst	Astragaloside A, Calycosin-7-Glucoside	848
Pilose Asiabell Root (*Dang Shen*)	*Codonopsis Radix*	Sweet, slightly sour, neutral	Supplements the center, boosts qi, fortifies the lung, boosts the spleen. Apply to weakness of spleen and lung, short breath and palpitations, anorexia and loose stool, dyspnea and cough due to deficiency of the lung, feverish dysphoria and diabetes	Spinaterol, Stigmasterol	816

^a^According to the Pharmacopoeia of the People’s Republic of China (2020 Edition)

**Table 4:** Summary of top five most frequently used patent proprietary CHM formulas

Chinese name in phonetic (Manufacturer)	Ingredients in phonetic	Actions	Indications	Adminstration and dosage	Frequency of usage
*Xi-Lei-San (Jiang Su 707 Natural Pharmaceutical Co. Ltd.)*	*Zhen Zhu, Qing Dai, Niu Huang, Xiang Ya Xie, Hua Shi, Bing Pian*	Promote anti-inflammatory, detoxification and granulation	It is indicated for ulcerative colitis that does not heal for a long time with the pattern of dampness-heat of large intestine	3 g q.d for external use enema	48
*Yun-Nan-Bai-Yao (Yun Nan Bai Yao Group Co. Ltd.)*	*San Qi, San Yu Cao, Chuan Shan Long, Huai Shan Yao, Lao Huan Cao, Bai Niu Dan, Ku Liang Jiang*	Resolve stasis, stop bleeding, activate blood, relieve pain, remove toxin, and disperse swelling	It is indicated for bleeding of ulcerative colitis	0.25–0.5 g q.i.d for oral use	18
*Fu-Fang-Ku-Shen-Jie-Chang-Rong-Jiao-Nang (Bei Jing Zhong Hui Pharmaceutical Co. Ltd.)*	*Ku Shen, Qing Dai, Di Yu, Bai Ji, Gan Cao*	Clear heat and dryness, detoxify and collect sore, cool blood and stop bleeding	It is indicated for ulcerative colitis caused by damp heat of the large intestine	1.6 g t.i.d for oral use	13
*Bu-Pi-Yi-Chang-Wan (Guang Zhou Bai Yun Mountain Chen Li Ji Pharmaceutical Co. Ltd.)*	*Huang Qi, Dang Shen, Bai Zhu, Sha Ren, Bai Shao, Rou Gui, Gan Jiang, Mu Xiang, Yan Hu Suo, Bu Gu Zhi, Chi Shi Zhi, Fang Feng*	Nourish qi and blood, warm *yang* qi, astringent bowel and antidiarrheal	It is indicated for ulcerative colitis that does not heal for a long time due to deficiency of spleen and stomach	6 g t.i.d for oral use	7
*Shen-Ling-Bai-Zhu-San (Yun Nan Bai Yao Group Co. Ltd.)*	*Ren Shen, Fu Ling, Bai Zhu, Shan Yao, Bai Bian Dou, Lian Zi, Yi Yi Ren, Sha Ren, Jie Geng, Gan Cao*	Tonify spleen and stomach, and invigorate lung qi	It is indicated for ulcerative colitis patients with indigestion or other symptoms caused by spleen-stomach weakness	6-9 g t.i.d/b.i.d for oral use	7

### CM *zheng*‑based CHM application and their relationships

**Fig. 4:**
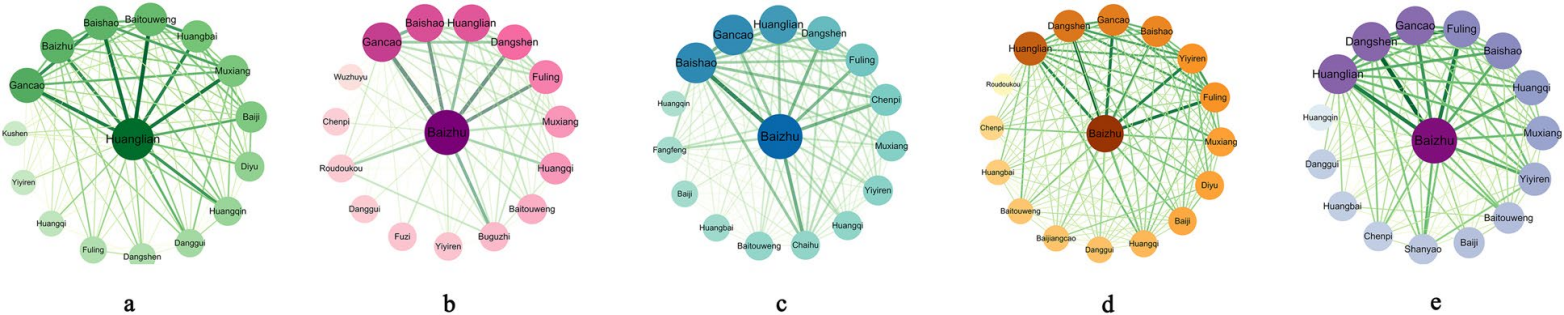
Network relationship of Chinese herbal medicines in the tretment of UC based on different CM patterns (**a** Large intestine dampness-heat; **b** Spleen-kidney yang deficiency; **c** Liver depression and spleen deficiency; **d** Spleen-stomach weakness; **e** Spleen deficiency with dampness accumulation.)

Among 895 individual studies including CM *zheng*, we identified a total of 43 different CM patterns (*zheng*) for UC and particularly listed the top ten in Table 5. The most common pattern was *Large intestine dampness-heat* (*Da Chang Shi Re*) (frequency = 517), followed by *Spleen-kidney yang deficiency* (*Pi Shen Yang Xu*) (frequency = 192), *Liver depression and spleen deficiency* (*Gan Yu Pi Xu*) (frequency = 154), *Spleen-stomach weakness* (*Pi Wei Xu Ruo*) (frequency = 143), and *Spleen deficiency with dampness accumulation* (*Pi Xu Shi Yun*) (frequency = 76). According to these top five CM *zheng* of UC, we analyzed the herbal application and their interactions based on each CM pattern (Fig. 4). Generally, the core herbs (the biggest node with the deepest colorful) of each CM *zheng* were *Huang Lian* for the pattern of *Large intestine dampness-heat*, and *Bai Zhu* for the other four patterns of *Spleen-kidney yang deficiency*, *Liver depression and spleen deficiency*, *Spleen-stomach weakness*, and *Spleen deficiency with dampness accumulation*. We also identified a total of fifteen commonly herbs which were closely related to the core herb of each pattern. The thicker lines with darker colorful represents the stronger correlation to the core herb in clinical application. For example, *Huang Lian* was closely related to *Bai Tou Weng*, *Huang Bai*, *Huang Qin*, *Bai Shao*, *Mu Xiang*, *Bai Ji*, *Bai Zhu* and *Gan Cao* for the treatment of *Large intestine dampness-heat*. *Bai Zhu*, as the core herb of other four CM *zheng*, was usually combined with *Dang Shen*, *Fu Ling*, *Gan Cao*, *Huang Lian* and *Mu Xiang*. But there were some differences in herbal compatibility for each CM pattern, which are presented in Fig. 4a–e.

**Table 5:** Top ten most commonly used CM pattern (*zheng*) for UC

Name	Therapeutic principle	Number of subjects diagnosed as CM *zheng*^a^	Frequency of reporting
*Large intestine dampness-heat* (*Da Chang Shi Re*)	Clear heat and dampness, harmony qi and blood	23,031	517
*Spleen-kidney yang deficiency* (*Pi Shen Yang Xu*)	Warm and tonify spleen and kidney	5585	192
*Liver depression and spleen deficiency* (*Gan Yu Pi Xu*)	Sooth liver and strengthen spleen	3849	154
*Spleen-stomach weakness* (*Pi Wei Xu Ruo*)	Tonify the spleen and stomach	2341	143
*Spleen deficiency with dampness accumulation* (*Pi Xu Shi Yun*)	Tonify spleen and resolve dampness	2148	76
*Qi stagnation and blood stasis* (*Qi Zhi Xue Yu*)	Regulate qi and activate blood	556	70
*Dampness-heat due to spleen deficiency* *(Pi Xu Shi Re)*	Tonify spleen and promote dehumidification	3087	61
*Cold and heat in complexity* *(Han Re Cuo Za)*	Clear heat and warm yang	2613	61
*Internal obstruction of stagnant blood* *(Yu Xue Nei Zu)*	Activate blood and remove stasis	312	44
*Deficiency of yin and blood* *(Yin Xue Kui Xu)*	Tonify yin and blood	120	44

^a^The caculation of this item is based on 616 clincial studies, not 895. Because among 895 clinical studies included CM pattern diagnosis, 616 trials included only one type of CM pattern for the inclusion of UC participants, while the remining 279 studies involed more than one type of CM patterns (e.g., for some studies, the UC participants were diagnosed as the combination of “*Large intestine dampness-heat*”, “*Spleen-kidney yang deficiency*” and “*Spleen-stomach weakness*”; for other studies, the subjects were diagnosed as the pattern of “*Liver depression and spleen deficiency*” or “*Spleen-stomach weakness*”, etc.). As the specific number of subjects for each pattern could not identified in these cases, we thereby excluded the 279 trials with complex patterns and caculated this item (number of subjects diagnosed as CM pattern) based on the 616 trials with single pattern

## Discussion

This study provides a comprehensive review of the characteristics of CHM treatments for UC in the past 30 years, which have identified a remarkable increase in the number of publications after 2007. In this review, we have identified that the most common studied condition(s), intervention(s) and comparison(s) of included articles were active UC, CHM fixed formula in decoction, and active control. Most included studies are RCT with design of parallel two arms, single centre, and open label. It is important to note that only 697 (30.2%) trials mentioned the safety assessment of CHM interventions, of which 230 (33.0%) articles reported the specific adverse effects (e.g., symptoms of the gastrointestinal tract, etc.). The reasons of there are less emphasized in safety consideration of clinical studies might be the mistakenly assumption of Chinese medicinal substances originating from natural sources are harmless [27]. However, adverse effects derive from unpredictable adverse events, improper use, contamination, misidentification, and herb-drug interactions [28]. Inadequate or absent reporting of safety evaluation on CHM interventions greatly degrades the scientific evidence of clinical trials. Therefore, we recommend that the future clinical studies should pay more attention to the high standard of study design, especially for the randomized, multicentre, double blind, placebo control (or double-dummy), and large sample size.

Moreover, although the CM pattern differentiation is critical in the determination of CHM interventions, there are only 895 (38.7%) studies that adopted the CM pattern diagnosis for the inclusion of UC participants and 524 (22.7%) articles that included CM-related outcomes, such as pattern scale questionnaire. In a CHM interventional trial, if the CM pattern is included, the concept of pattern differentiation should be carried out throughout the entire process with regard to the rationale of the study design, selection of inclusion and exclusion criteria, CHM formula prescription, selection of outcome measures and data interpretation, etc. In clinical practice, the CHM treatment is guided with CM principles and determined by pattern differentiation. If the pattern of a condition is misdiagnosed, the treatment principles will be incorrect, and its derivative CHM formula will be ineffective. Therefore, we recommend that the reporting of such studies should follow the guideline of “CONSORT Extension for Chinese Herbal Medicine Formulas 2017: Recommendations, Explanation, and Elaboration", especially for the reporting of details and rationale regarding the definite diagnostic criteria of studied CM pattern, correspondence between pattern identification and CHM formula (e.g., *Fang-Zheng-Dui-Ying*), and CM-related outcome(s) [29, 30].

In this review, we systematically examined the use of single herbs, CHM fixed formulas, individualized formulas and patent proprietary formulas, and the combination of CHM and Western medicine in the clinical practice of UC. We identified a total of 245 types of CHM formulas and further summarized the top five frequently used formulas and top ten commonly herbs. Specifically, the classic CHM formula included *Bai-Tou-Weng-Tang*, *Shen-Ling-Bai-Zhu-San*, *Shao-Yao-Tang*, *Bu-Zhong-Yi-Qi-Tang*, and *Tong-Xie-Yao-Fang*. Sulfasalazine and Mesalazine are the most common concomitant western medicines for CHM formulas. The patent proprietary CHM formulas are *Xi-Lei-San* (enema), *Yun-Nan-Bai-Yao*, *Fu-Fang-Ku-Shen-Jie-Chang-Rong-Jiao-Nang*, *Bu-Pi-Yi-Chang-Wan*, and *Shen-Ling-Bai-Zhu-San*. It is indicated that the administration route of CHM interventions for UC not only include oral delivery, but also have the external use with directly effect, such as enema. The most common herbs in practice mainly focused on the types of heat-clearing, dampness-draining, toxin-removing, spleen-invigorating, qi-moving, and blood-stopping, such as *Huang Lian*, *Huang Bai*, *Bai Tou Weng*, *Bai Zhu*, *Gan Cao*, *Bai Shao*, *Huang Qi*, *Dang Shen*, *Mu Xiang*, and *Bai Ji*. More than half of these identified CHM therapies are consistent with the recommendations of “Experts-based consensus of integrative Chinese and western medicine treatment for ulcerative colitis (2017 version)” [31]. Moreover, the results also provide the basis and reference for the future update of clinical practice guideline for UC in terms of CM diagnosis and treatment.

According to CM theory, *zheng* (pattern) is a pathological cluster or summary of signs and symptoms at a certain stage of a disease, which reflect the relationship between the pathogens and the body’s resistance [32]. Practically, pattern identification refers to the analysis and summarization of the clinical symptoms obtained through the four diagnostic methods of CM (inspection, auscultation and smell, inquiry, and pulse taking and palpation), after which CM practitioners can accordingly determine the therapeutic principles and select the appropriate CHM treatments based on the patient’s current essential pattern [33, 34]. In this study, we found that the most common CM *zheng* of UC patients were the patterns of *Large intestine dampness-heat*, *Spleen-kidney yang deficiency*, *Liver depression and spleen deficiency*, *Spleen-stomach weakness*, and *Spleen deficiency with dampness accumulation*, which reflected the progress of UC with different phases. Through the association analysis on herbal applications and their relationships, we formulated one core herb with fifteen commonly combined herbs for each CM pattern. Specifically, in the CM pattern of *Large intestine dampness-heat*, *Huang Lian*, a typical heat-clearing and dampness-drying medicinal, was more frequently combined with the following three categories of herbs: (i) dispelling and heat-clearing medicinal, including *Bai Tou Weng*, *Huang Bai*, and *Huang Qin*; (ii) harmonizing qi and blood medicinal, including *Bai Shao*, *Mu Xiang*, *Dang Gui*, and *Bai Ji*; and iii) tonifying qi and invigorating spleen medicinal, including *Bai Zhu* and *Gan Cao*. Among these, the compatibility of blood-activating and qi-moving medicinals was very important due to the identification of strong correlations between *Mu Xiang* and *Bai Shao*/*Gan Cao*. Besides, herbs of invigorating spleen and draining dampness (e.g., *Dang Shen*, *Huang Qi*, *Fu Ling*, *Yi Yi Ren*) and astringent hemostatic (e.g., *Di Yu*) could be supplemented in the CHM formula for the treatment of UC with *Large intestine dampness-heat* pattern (Fig. 4a).

For the remaining four CM patterns, *Bai Zhu*, as their common core herb, was usually combined with *Dang Shen*, *Fu Ling*, and *Gan Cao* to treat the pattern of *spleen deficiency* which is the basic type included in the four patterns. In addition, *Huang Lian* and *Mu Xiang* were commonly added in the prescriptions to treat the four patterns, due to their role of heat-clearing, dampness-drying, and qi-moving. However, there are some differences among each specific pattern. In terms of *Spleen-kidney yang deficiency*, herbs of warming and tonifying *yang* qi, astringing intestines and checking diarrhea were emphasized, including *Bu Gu Zhi*, *Rou Dou Kou*, *Wu Zhu Yu*, and *Huang Qi* (Fig. 4b). For the pattern of *Liver depression and spleen deficiency*, *Bai Shao* is another vital herb, similar to the core herb of *Bai Zhu*, which was associated with *Chai Hu*, *Fang Feng* and *Chen Pi* to achieve soothing liver, emolliating liver, regulating qi and relieving depression (Fig. 4c). Compared the two patterns of *Spleen-stomach weakness* and *Spleen deficiency with dampness accumulation*, the former presented more correlations between the core herb and tonifying qi and invigorating spleen medicinal, such as *Shan Yao* and *Huang Qi* (Fig. 4d); the latter focused on the draining or drying dampness medicinal, such as *Yi Yi Ren*, and added more herbs for clearing heat, removing toxin, cooling blood, and stopping bleeding, such as *Di Yu*, *Bai Ji*, *Bai Jiang Cao*, and *Bai Tou Weng* (Fig. 4e). Based on the results of herbal correlation analysis, the CM treatment principles for UC could be summarized as clear heat and dampness, tonify and strength spleen, and harmony qi and blood.

This study has some limitations. First, this review identified articles published up to 25 November 2020 in the targeted seven databases. Any records which had not been included in these databases by that cut-off period have not been included. In addition, we included only articles in English and Chinese because of language limitations. As such, we may not have captured otherwise eligible trials published in other languages. Second, the data were extracted from clinical studies that used seven different types of design, including both interventional and observational studies (e.g., RCTs, cohort study, case–control study, etc.). The quality of these studies varied and therefore it is difficult to compare them quantitatively. Thus, we only provided the descriptive analysis for the general characteristics of included studies, not included the methodology and reporting quality assessments.

## Conclusion

This review examined the application of CHM interventions for UC and summarized the 10 most frequently used single herbs, the 5 most frequently used CHM formulas and patent proprietary formulas, and the combined use of CHM and Western medicine treatments. The characteristics of CM pattern identification in UC and their herbal prescription in practice were also analyzed.

## Supplementary Information

**Additional file 1: Appendix S1.** Search Strategy. **Appendix S2.** Correction script for data check. **Appendix S3.** Reported adverse effects of included studies. **Appendix S4.** Full name list of summarized CHM formulas.

## Data Availability

The data used for this study are included in the manuscript and additional file.

## Abbreviations

UC: Ulcerative colitis
CHM: Chinese herbal medicines
CAM: Complementary and alternative medicine
CM: Chinese medicine
WM: Western Medicine
RCTs: Randomized controlled trials
EBM: Evidence-Based Medicine
AMED: Allied and Complementary Medicine
CNKI: China National Knowledge Infrastructure
ARM: Association Rule Mining
