# Supplementary material for: Chinese herbal medicines in the treatment of ulcerative colitis: a review
Source: Chin Med. 2022 Apr 4;17:43. doi: 10.1186/s13020-022-00591-x (PMC8981751; doi:10.1186/s13020-022-00591-x)
Supplement: Supplementary file 1 — Additional file 1: Appendix S1. Search Strategy. Appendix S2. Correction script for data check. Appendix S3. Reported adverse effects of included studies. Appendix S4. Full name list of summarized CHM formulas. [file 13020_2022_591_MOESM1_ESM.pdf]

Appendix 1. Search Strategy

Search for English Databases: EBM Reviews - Cochrane Database of Systematic Reviews <2005 to November 19, 2020>, EBM Reviews - ACP Journal Club <1991 to October 2020>, EBM Reviews - Database of Abstracts of Reviews of Effects <1st Quarter 2016>, EBM Reviews - Cochrane Clinical Answers <November 2020>, EBM Reviews - Cochrane Central Register of Controlled Trials <October 2020>, EBM Reviews - Cochrane Methodology Register <3rd Quarter 2012>, EBM Reviews - Health Technology Assessment <4th Quarter 2016>, EBM Reviews - NHS Economic Evaluation Database <1st Quarter 2016>, **AMED** (Allied and Complementary Medicine) <1985 to October 2020>, **Embase** <1974 to 2020 November 25>, Ovid **MEDLINE**(R) <1946 to November 25, 2020>

Search Strategy:

1 (colitis and ulcerat*).mp.
2 ulcerative colitis.mp. or exp ulcerative colitis/
3 (inflammatory bowel disease* or IBD).mp.
4 or/1-3
5 random$.tw.
6 factorial$.tw.
7 (crossover$ or cross over$ or cross-over$).tw.
8 placebo$.tw.
9 single blind.mp.
10 double blind.mp.
11 triple blind.mp.
12 (singl$ adj blind$).tw.
13 (double$ adj blind$).tw.
14 (tripl$ adj blind$).tw.
15 assign$.tw.
16 allocat$.tw.
17 randomized controlled trial/
18 or/5-17
19 exp cohort studies/
20 exp case-control studies/
21 exp retrospective studies/
22 exp Epidemiologic Studies/
23 case-control studies/ or longitudinal studies/ or follow-up studies/ or prospective studies/ or cross-sectional studies/
24 (cohort$ or prospective$ or retrospective$).mp.
25 or/19-24
26 18 or 25
27 (chinese medicine or herb$).mp.
28 [exp Medicine, Traditional/] {Including Limited Related Terms}
29 ethnobotany.mp. or exp Ethnobotany/
30 phytotherapy.mp. or exp Phytotherapy/

31 Plant Extracts.mp. or exp Plant Extracts/
32 medicinal plant.mp. or exp Plants, Medicinal/
33 chinese herbal drugs.mp. or exp Drugs, Chinese Herbal/
34 chinese traditional medicine.mp. or exp Medicine, Chinese Traditional/
35 herbal medicine.mp. or exp Herbal Medicine/
36 (medicinal herb or pharmaceutical plant).mp.
37 (Chinese medicine$ or traditional medicine$).mp.
38 (herbal drug$ or herbal medicine$).mp.
39 (medicinal plant$ or medicinal herb$).mp.
40 (herb$ or herb$ formula$ or decoction$).mp.
41 (herb$ granule$ or herb$ capsule$ or herb$ pellet$).mp.
42 materia medica.mp. or exp Materia Medica/
43 or/27-42
44 ((single#entity or single) adj3 (component or drug$ or herb$)).mp.
45 (compound prescription$ or herbal mixture or Fufang).mp.
46 (Chinese Medicine Patent Prescription or proprietary Chinese medicines).mp.
47 (Chinese patent adj3 (medicine or drug$)).mp.
48 (Chinese adj3 (patent or proprietary) adj3 (medicine or drug$)).mp.
49 (Chinese adj2 (patent or proprietary) adj2 (medicine or drug$ or prescription$)).mp.
50 or/44-49
51 43 or 50
52 4 and 26 and 51

Search for Chinese Databases:

Search strategy for **CNKI**:
SU=('临床试验'+'随机'+'对照'+'随机试验'+'随机对照试验'+'临床研究'+'半随机'+'队列'+'病例对照') and SU=('溃结'+'溃疡性结肠炎'+'炎症性肠病'+'炎性肠疾病'+'慢性非特异性溃疡性结肠炎') and SU=('中医药'+'草药'+'中药'+'复方'+'汤剂'+'中成药'+'注射剂'+'加减方'+'外用中药')

Search strategy for **VIP**:
(M=临床试验+M=随机+M=对照+M=随机试验+M=随机对照试验+M=临床研究+M=半随机+M=队列+M=病例对照)*(M=溃结+M=溃疡性结肠炎+M=炎症性肠病+M=炎性肠疾病+M=慢性非特异性溃疡性结肠炎)*(M=中医药+M=草药+M=中药+M=复方+M=汤剂+ M=中成药+M=注射剂+M=加减方+M=外用中药)

Search strategy for **Wanfang**:
(“临床试验”or”随机”or”对照”or”随机试验”or”随机对照试验”or”临床研究” or”半随机”or”队列”or”病例对照”)and(“溃结”or”溃疡性结肠炎”or”炎症性肠病”or”炎性肠疾病”or”慢性非特异性溃疡性结肠炎”)and (“中医药”or”草药”or”中药”or”复方”or”汤剂”or”中成药”or”注射剂”or”加减方”or”外用中药”)

Appendix 2. Correction script for data check

```
x = 1
Do While x < 450:
    x = x + 1
    ActiveCell.Offset(1, 0).Range("A1").Select
    Range(Selection, Selection.End(xlDown)).Select
    Application.CutCopyMode = False
    Selection.Copy
    ActiveCell.Offset(0, -1).Range("A1").Select
    Selection.End(xlDown).Select
    ActiveCell.Offset(1, 0).Range("A1").Select
    ActiveSheet.Paste
    ActiveCell.Offset(0, 1).Range("A1").Select
    Selection.End(xlUp).Select
    Selection.End(xlUp).Select
    Application.CutCopyMode = False
    Selection.Copy
    Selection.End(xlToLeft).Select
    Selection.End(xlDown).Select
    ActiveCell.Offset(1, 0).Range("A1").Select
    ActiveSheet.Paste
    ActiveWindow.SmallScroll Down:=21
    Application.CutCopyMode = False
    Selection.AutoFill Destination:=ActiveCell.Range("A1:A72"), Type:= _
        xlFillDefault
    ActiveCell.Range("A1:A72").Select
    ActiveCell.Offset(0, 1).Range("A1").Select
    Selection.End(xlDown).Select
    ActiveCell.Offset(1, -1).Range("A1").Select
```

```
    Range(Selection, Selection.End(xlDown)).Select
    Selection.ClearContents
    ActiveCell.Offset(0, 2).Range("A1").Select
    Selection.End(xlUp).Select
    Selection.End(xlUp).Select
    ActiveCell.Columns("A:A").EntireColumn.Select
    Selection.Delete Shift:=xlToLeft
    Selection.End(xlUp).Select
    Loop
End Sub
```

Appendix 3. Reported adverse effects of included studies

Specifics	Frequency
Nausea	144
Rash	41
Abdominal distension	39
Abnormal liver and renal function	32
Headache	29
Loss of appetite	26
Abdominal Pain	25
Vomiting	23
Anal irritation	17
Dizzy	14
Constipation	12
Diarrhea	12
Leukopenia	8
Fatigue	7
Dry mouth	6
Sore throat	6
Fever	5
Infection	5
Bitter mouth	4
Menstrual abnormalities	4
Insomnia	3
Heart palpitations	3
Joint pain	3
Edema	2
Back pain	2
Bone marrow suppression	1
Anxiety	1
Cushing's syndrome	1
Dark urine	1
Anemia	1
Drowsiness	1
Alopecia	1
Hypertension	1

Appendix 4. Full name list of summarized CHM formulas

Chinese name in phonetic	Frequency
Bai-Tou-Weng-Tang	466
Can-Ling-Bai-Shu-San	276
Shao-Yao-Tang	180
Bu-Zhong-Yi-Qi-Tang	139
Tong-Xie-Yao-Fang	133
Si-Shen-Wan	131
Si-Jun-Zi-Tang	88
Wu-Mei-Wan	81
Ge-Gen-Qin-Lian-Tang	79
Ban-Xia-Xie-Xin-Tang	53
Xi-Lei-San	48
Li-Zhong-Tang	46
San-Huang-Tang	40
Xiao-Yao-San	33
Fu-Zi-Li-Zhong-Tang	29
Liu-Jun-Zi-Tang	24
Shi-Xiao-San	18
Yun-Nan-Bai-Yao	18
Zhen-Ren-Yang-Zang-Tang	16
Chai-Hu-Shu-Gan-San	14
Fu-Fang-Huang-Bai-Ye	13
Fu-Fang-Ku-Can-Jie-Chang-Rong-Jiao-Nang	13
Xiang-Sha-Liu-Jun-Zi-Tang	12
Huang-Lian-Jie-Dong-Tang	11
Zhu-Che-Wan	11
Shao-Fu-Zhu-Yu-Tang	10
Si-Ni-Tang	10
Chai-Shao-Liu-Jun-Tang	9
Si-Wu-Tang	8
Bu-Pi-Yi-Chang-Wan	7
Da-Huang-Huang-Lian-Xie-Xin-Tang	7
Lian-Li-Tang	7
Xiang-Lian-Wan	7
Si-Ni-San	7
Huai-Hua-San	7
Dang-Gui-Shao-Yao-San	6
Ba-Wei-Xi-Lei-San	6
Yi-Yi-Fu-Zi-Bai-Jiang-San	6
Ge-Gen-Qin-Lian-Wu-Tan-Tang	6
Tao-Hua-Tang	6
Huang-Qin-Tang	6
Xiao-Chai-Hu-Tang	5

Sheng-Mai-San	5
Xue-Fu-Zhu-Yu-Tang	4
Sheng-Yang-Yi-Wei-Tang	4
Liu-Wei-Di-Huang-Wan	4
Huang-Lian-A-Jiao-Tang	4
Ge-Xia-Zhu-Yu-Tang	4
Fu-Zi-Li-Zhong-Wan	4
Liu-He-Tang	4
Xian-Fang-Huo-Ming-Yin	3
Wu-Mei-Bai-Jiang-Fang	3
Tao-Hong-Si-Wu-Tang	3
Qin-Bai-Ke-Li-Ji	3
Qi-Qi-Gu-Ben-Tang	3
Huo-Xiang-Zheng-Qi-San	3
Gan-Cao-Xie-Xin-Tang	3
Dang-Gui-Si-Ni-Tang	3
Chang-Wei-Qing-Jiao-Nang	3
Chai-Hu-Shao-Yao-Tang	3
Wen-Jing-Tang	3
Gui-Pi-Tang	3
Chang-Di-Qing	3
Ba-Zhen-Tang	3
Xiao-Jian-Zhong-Tang	3
Yun-Nan-Bai-Yao-Jiao-Nang	3
Jiao-Dai-Hu-Ji	3
Jian-Pi-He-Ji	3
Huang-Lian-Jian-Ji	3
Xue-Jie-Fen	3
Qing-Chang-Shuan	3
Lu-Dong-San	3
Li-Zhong-Wan	3
Ju-Yuan-Jian	3
Dong-Miao-San	3
Xiang-Sha-Yang-Wei-Wan	2
Tong-Fu-Ning-Ke-Li	2
Shi-Quan-Da-Bu-Tang	2
Sheng-Ma-Ge-Gen-Tang	2
Shen-Tong-Zhu-Yu-Tang	2
Shao-Yao-Gan-Cao-Tang	2
Qi-Wei-Bai-Shu-San	2
Ma-Chi-Jian-Jiao-Nang	2
Liu-Wei-Di-Huang-Tang	2
Kui-Jie-Kang-Jiao-Nang	2
Jin-Gui-Shen-Qi-Wan	2

Gu-Chang-Zhi-Xie-Wan	2
Gu-Ben-Yi-Chang-Pian	2
Ge-Gen-Ling-Lian-Tang	2
Diao-Wei-Cheng-Qi-Tang	2
Diao-Chang-Xiao-Yan-Pian	2
Dan-Zhi-Xiao-Yao-San	2
Chang-Yan-Ning-Jiao-Nang	2
Chang-Te-Ling-Shui-Wan	2
A-Jiao-Mei-Lian-Wan	2
Fu-Fang-Dan-Can-Zhu-She-Ye	2
Dan-Xiang-Guan-Xin-Zhu-She-Ye	2
Yang-He-Tang	2
Wen-Pi-Tang	2
Wei-Ling-Tang	2
Sheng-Yu-Tang	2
Liu-He-Tang	2
Huang-Tu-Tang	2
Zhi-Kang-Jiao-Nang	2
Huang-Qin-Tang-Ke-Li-Ji	2
Bai-Shao-Qi-Wu-Ke-Li	2
Zuo-Jin-Wan	2
Yi-Gong-San	2
San-Qi-Fen	2
Jian-Pi-Shuan	2
Dan-Can-Fen	2
Bai-Ji-Fen	2
Yu-Ping-Feng-San	2
Long-Xue-Jie-San	2
Bu-Huan-Jin-Zheng-Qi-San	2
Zhu-She-Yong-Dan-Can	1
Zhi-Shi-Dao-Zhi-Tang	1
Zhi-Zi-Bai-Pi-Tang	1
Yu-Ping-Feng-Ke-Li	1
Yu-Dong-Xiao-Jiao-Nang	1
Xi-Jiao-Di-Huang-Tang	1
Wu-Mei-Bai-Jiang-Tang	1
Tong-Xie-Ning-Ke-Li	1
Tao-Ren-Cheng-Qi-Tang	1
Shu-Gan-Li-Pi-Pian	1
Sheng-Mai-Zhu-She-Ye	1
Shao-Yao-Si-Ni-Tang	1
Mu-Xiang-Bing-Lang-Wan	1
Long-Xue-Jie-Jiao-Nang	1
Ling-Gui-Shu-Gan-Tang	1

Lian-Qiao-Bai-Dong-San	1
Kui-Jie-Ling-Ke-Li	1
Jiu-Xie-Ling-Chong-Ji	1
Jie-Chang-Ling-Jiao-Nang	1
Jie-Chang-Kang-Fu-Wan	1
Jie-Chang-An-Jiao-Nang	1
Jiang-Huang-Shui-Jian-Ji	1
Jian-Pi-Hua-Zhi-Wan	1
Jian-Pi-Hua-Shi-Tang	1
Huo-Xue-Zhu-Yu-Tang	1
Huang-Qi-Jian-Zhong-Tang	1
Hou-Pu-Wen-Zhong-Tang	1
Hong-Hua-Zhu-She-Ye	1
Gu-Sui-Bu-Jian-Ji	1
Gan-Jiang-Fen-Jiao-Nang	1
Gan-Wei-Bai-He-Tang	1
Gan-Cao-A-Jiao-Tang	1
Fu-Fang-Gan-Cao-Pian	1
Fu-Fang-Dan-Can-Pian	1
Dong-Cha-Shui-Jian-Ji	1
Dan-Can-Dong-Gan-Fen	1
Da-Huang-Fu-Zi-Tang	1
Bu-Zhong-Yi-Qi-Wan	1
Bu-Yang-Huan-Wu-Tang	1
Bu-Pi-Yi-Shen-Wan	1
Bai-Jiang-Ku-Can-Tang	1
Ba-Wei-Di-Huang-Tang	1
Ma-Huang-Fu-Zi-Xi-Xin-Tang	1
Huang-Qi-Gui-Zhi-Wu-Wu-Tang	1
Fu-Fang-Xue-Jie-Guan-Chang-Ye	1
Fu-Fang-Ku-Can-Zhu-She-Ye	1
Fu-Zi-Yi-Yi-Bai-Jiang-San	1
Da-Huang-Fu-Zi-Xi-Xin-Tang	1
Chi-Shi-Zhi-Yu-Yu-Liang-Tang	1
Chai-Hu-Gui-Zhi-Gan-Jiang-Tang	1
Ban-Lan-Gen-Mian-Jian-Ke-Li	1
Chang-Kang-Ning-Jie-Chang-Ba-Xiang-Jiao-Nang	1
Huang-Qi-Jian-Zhong-Tang	1
Xiao-Ai-Ping-Zhu-She-Ye	1
Ci-Wu-Jia-Zhu-She-Ye	1
Chang-Yan-Kang-Kou-Fu-Ye	1
Yu-Xing-Cao-Zheng-Liu-Guan-Chang-Ye	1
Xian-Jie-Tang	1
Wen-Dan-Tang	1

Si-Jun-Tang	1
Shun-Qi-Tang	1
Sheng-Hua-Tang	1
Liu-Mo-Tang	1
Kui-Jie-Ning	1
Jie-Chang-Kang	1
Huang-Qi-Tang	1
Gui-Shao-Tang	1
Ge-Gen-Tang	1
Di-Yu-Tang	1
Dan-Can-Tang	1
Da-Huang-Tang	1
Chang-Yan-Kang	1
Bai-Shu-Tang	1
Yin-Chen-Hao-Tang	1
Xue-Jie-Jiao-Nang	1
Xiao-Qing-Long-Tang	1
Sheng-Jiang-Jiao-Nang	1
Qian-Kun-Chang-Kang	1
Hu-Di-Jiao-Nang	1
Da-Cheng-Qi-Tang	1
Chang-An-Jiao-Nang	1
An-Chang-Jiao-Nang	1
Shuang-Bai-Jian-Pi-Jiao-Nang	1
Qi-Wei-Wen-Shen-Jiao-Nang	1
Hu-Di-Chang-Rong-Jiao-Nang	1
Gu-Can-Chang-An-Jiao-Nang	1
Fu-Fang-Qing-Dai-Jiao-Nang	1
Fu-Fang-Da-Huang-Jiao-Nang	1
Ba-Wei-Zhi-Xie-Jiao-Nang	1
Qi-Can-Chang-Tai-Chang-Rong-Jiao-Nang	1
Ya-Dan-Zi	1
Kui-Jie-He-Ji	1
Luo-Hua-Zi-Zhu-Ke-Li	1
Kui-Jie-Kang-Guan-Chang-Ji	1
Zuo-Gui-Wan	1
Zi-Yu-Shuan	1
Zang-Lian-Wan	1
Yu-Bai-San	1
Yi-Guan-Jian	1
Xiao-Yao-Wan	1
Wu-Ji-Wan	1
Wu-Ling-San	1
Wu-Jin-San	1

Si-Shen-Pian	1
Shi-Hui-San	1
San-Huang-Wan	1
Qing-Bai-Shuan	1
Qian-Xi-Pian	1
Qi-Lian-Pian	1
Nuan-Gan-Jian	1
Ma-Chi-Jian	1
Liu-Shen-Wan	1
Liang-Ge-San	1
Kui-Yang-San	1
Kui-Jie-Shuan	1
Jian-Pi-Wan	1
Gui-Pi-Wan	1
Dan-Can-Pian	1
Chang-Yan-Shuan	1
Bing-Peng-San	1
Wu-Bei-Zi-San	1
Jin-Qiao-Mai-Pian	1
Jie-Chang-Ning-Wan	1
Fu-Fang-Dan-Can	1
Di-Jin-Cao-Pian	1
Dan-Can-Fen-Zhen	1
Chang-Yan-Qing-Pian	1
Xiang-Sha-Liu-Jun-Zi-Wan	1
Can-Qi-Wu-Wei-Zi-Pian	1
Ya-Dan-Zi-You	1
